# Association of Adiponectin Gene Polymorphism with Nonalcoholic Fatty Liver Disease in Taiwanese Patients with Type 2 Diabetes

**DOI:** 10.1371/journal.pone.0127521

**Published:** 2015-06-04

**Authors:** Ching-Jung Hsieh, Pei Wen Wang, Tsung Hui Hu

**Affiliations:** 1 Division of Endocrinology and Metabolism, Department of Internal Medicine, Kaohsiung Chang Gung Memorial Hospital and Chang Gung University College of Medicine, Kaohsiung, 123, Ta-Pei Road, Niao-Sung District, Kaohsiung City, Taiwan; 2 Hepatogastroenterology, Department of Internal Medicine, Kaohsiung Chang Gung Memorial Hospital and Chang Gung University College of Medicine, Kaohsiung, 123, Ta-Pei Road, Niao-Sung District, Kaohsiung City, Taiwan; National Taiwan University College of Medicine, TAIWAN

## Abstract

**Objective:**

Patients with type 2 diabetes and nonalcoholic fatty liver disease (NAFLD) have a higher prevalence of cardiovascular diseases. In this study we investigated the frequency of single nucleotide polymorphisms (SNPs) of several candidate genes associated with NAFLD in Taiwanese patients with type 2 diabetes mellitus (DM) and NAFLD and in those with DM but without fatty liver disease.

**Methods:**

We enrolled 350 patients with type 2 DM and NAFLD and 209 patients with DM but without NAFLD. Body mass index (BMI), % body fat (% BF), glycated hemoglobin (HbA1c), high molecular weight (HMW) isoform of adiponectin, aspartate aminotransferase (AST), alanine aminotransferase (ALT), total cholesterol (TC), low-density lipoprotein (LDL), high-density lipoprotein (HDL), and triglyceride (TG) levels were measured. Thirteen SNPs in 5 genes (adiponectin, leptin, peroxisome proliferator-activated receptor alpha, adiponutrin/patatin-like phospholipase domain-containing protein 3 and peroxisome proliferator-activated receptor γ co-activator 1α ) were measured.

**Results:**

Only adiponectin rs266729 polymorphism was associated with susceptibility to NAFLD (p = 0.001). Subgroup analysis revealed that the proportion of subjects with homozygous genotype GG was higher in patients with NAFLD (31%) than in controls (11%) and that the proportions of heterozygous CG and homozygous CC were higher in controls (37% and 52%, respectively) than in patients with NAFLD (33% and 36%, respectively). Patients with NAFLD carrying the GG genotype of rs266729 showed significantly lower serum HMW adiponectin levels than patients carrying the GC or CC genotype (3.75±0.37 vs. 3.99±0.66 vs. 4.79±0.58 μg/ml, p< 0.001). Body fat and serum HMW adiponectin levels were the strongest predictors of developing NAFLD (p < 0.001 and 0.004, respectively).

**Conclusions:**

In patients with type 2 diabetes gene polymorphism of adiponectin rs266729 is associated with risk of NAFLD. G allele of rs266729 is associated with hypoadiponectinemia. Low serum adiponectin level may precipitate liver steatosis in patients with type 2 diabetes.

## Introduction

Nonalcoholic fatty liver disease (NAFLD) is the most common cause of chronic liver disease in many countries. NAFLD is the hepatic manifestation of metabolic syndrome and is associated with obesity, type 2 diabetes mellitus, dyslipidemia, high blood pressure, insulin resistance and cardiovascular disease [1–3]. NAFLD affects approximately 10–30% of the general population and 70–90% of people with type 2 diabetes mellitus (DM) [4–8]. Patients with type 2 diabetes and NAFLD have a higher prevalence of coronary, cerebrovascular and peripheral vascular diseases than patients with diabetes but without NAFLD [9,10]. The 2011 National Diabetes Fact Sheet revealed that more than 65% of all deaths in patients with type 2 diabetes are caused by cardiovascular disease. Adams *et al*. have also shown that cardiovascular disease accounts for about 25% of deaths in patients with NAFLD versus 13% of deaths in patients with other liver diseases [11]. Therefore, NAFLD may result in an increased risk of mortality, especially due to cardiovascular disease, in patients with type 2 diabetes.

The interaction of environmental and genetic factors can result in an NAFLD phenotype and influence its progression. Many single nucleotide polymorphisms (SNPs) in genes encoding proteins involved in the pathogenesis of NAFLD and type 2 diabetes may contribute to the progression of NAFLD. Many studies have revealed that genetic factors predispose an individual to NAFLD or type 2 diabetes and have led to the development of noninvasive biomarkers for early diagnosis of type 2 diabetes complicated with NAFLD, such as adiponectin, leptin, peroxisome proliferator-activated receptor alpha (PPAR-α), adiponutrin/patatin-like phospholipase domain-containing protein 3 (PNPLA3), peroxisome proliferator-activated receptor γ (PPAR-γ) and Peroxisome proliferator-activated receptor γ co-activator 1α (PPARGC1A) [12–19]. Genes related to insulin resistance including those coding for adiponectin, resistin, leptin, adiponutrin and PPAR-r have been suspected to influence the progression of NAFLD [20]. However, the pathogenesis of NAFLD development in patients with diabetes remains unclear.

Several common alleles have been identified as NAFLD risk factors in Asians and have been shown to be common in the pathogenesis of metabolic syndrome and NAFLD [21]. The SPN rs2290602 of the PPARGC1A gene has been shown to be associated with NAFLD in Japanese [15]. Lu et al found that the SNP rs1805096 of the LEPR gene probably contributes to the onset of NAFLD in patients with type 2 diabetes [13]. Variants of LEPR rs1137100 and rs1137101 have been shown to be associated with risk of NAFLD [14]. PPAR-alpha val227ala (rs 1800234) was shown to be involved in the pathogenesis of NAFLD and to be a protective factor against obesity in Chinese [16]. In Taiwanese children and in a Chinese patient group, PNPLA3 rs738409 has been reported to be associated with an increased risk of NAFLD [18,19]. In this study we investigated the frequency of SNPs of several candidate genes associated with NAFLD in Taiwanese DM patients with and without NAFLD.

## Materials and Methods

### Subjects

We enrolled 350 patients (187 women and 163 men, aged 52.0 ± 9.9 years) with NAFLD and 209 controls (123 women and 86 men, aged 51.4 ± 10.6 years) from the metabolism/endocrinology clinic at the Kaohsiung Chang Gung medical center, Taiwan. All subjects had type 2 diabetes for more than 1 year, received oral hypoglycemic agents, and had received more than one abdominal ultrasonographic examination within one year before participating in the study. Patients with NAFLD had an elastography-confirmed liver fat content > 30%. Elastography was performed using a FibroScan 502 device equipped with an M probe (Echosens, Paris, France) to capture both the controlled attenuation parameter (CAP) and liver stiffness measurement values simultaneously [22]. NAFLD was diagnosed based on a CAP > 227 dB/m/MHz. Subjects included in this study were more than 40 years old, ethnically Chinese, and from the same region in Taiwan at the commencement of the study. Exclusion criteria included pregnancy, chronic alcohol drinking, and hepatitis B or C carrier status as determined by taking a history and measuring hepatitis B surface antigen and anti- hepatitis C antibody. Patients with type 1 diabetes mellitus, those with type 2 diabetes taking thiazolidinedione or receiving insulin therapy within 1 year, and patients with secondary diabetes were also excluded. Chronic alcohol drinking was defined as the consumption of more than 15 grams of pure alcohol per day, as confirmed by a family member. Clinical and laboratory parameters including body mass index (BMI), % body fat (% BF), fasting plasma glucose, fasting insulin, glycated hemoglobin (HbA1c), high molecular weight (HMW) iso-form of adiponectin, aspartate aminotransferase (AST), alanine aminotransferase (ALT) and lipid profile including total cholesterol (TC), low-density lipoprotein (LDL), high-density lipoprotein (HDL), and triglyceride (TG) were measured.

### Ethics Statement

Subjects provided written informed consent after a full explanation of the research outline. The study protocol was reviewed and approved by the Medical Ethics Committee of the Chang Gung Memorial Hospital.

### Methods

BMI was calculated as body weight (kg)/ body height (m^2^). Patients were requested to wear light clothing during the measurements of weight and height. Total body fat mass was measured by electrical bioimpedance (Model 310, Body Composition Analyzer, BioDynamic, USA). All subjects were tested after a six-hour fast (including abstinence of food and water) while wearing light clothing and after empting the urinary bladder.

Fasting blood glucose, insulin, AST, ALT, HMW adiponectin levels and blood lipid profile including TC, LDL, HDL, and TG were checked after fasting for more than 10 hours. The concentrations of TC, LDL, HDL, TG, AST, and ALT were measured using an autoanalyzer (Hitachi 7250 Special; Hitachi, Tokyo, Japan). The HbA1c level was measured by high-pressure liquid chromatography (Bio-Rad Laboratories, Inc, Richmond, CA, USA). We measured plasma concentrations of glucose and insulin (Access automated immunoassay; Beckman Instruments, Fullerton, CA) at the same time. Insulin resistance (IR) was determined by homeostasis model assessment (HOMA) and calculated using fasting plasma glucose and fasting insulin levels in each participant, as follows:

HOMA IR = fasting glucose (mmol/l) × fasting insulin (mU/ml)/22.5.
HMW adiponectin levels were checked with commercially available ELISA kits (ALPCO Diagnostics; Salem, NH, USA) according to the manufacturer’s protocol. All samples were measured in duplicate. The detection limit is 0.019 μg/ml.

We selected 13 SNPs in 5 candidate genes from published literature and the Database of Single Nucleotide Polymorphism (dbSNP) at the NCBI website (http://www.ncbi.nlm.nih.gov/SNP), The SNPs of the candidate genes have all been detected in Asian populations.

- **PPARPGC1A:** rs3736265 (1835C>T), rs2290602 (+171A>C), rs3755863 (1584G>A)
- **Leptin receptor (LEPR)**: rs1805096 (3057G>A), rs1137101 (668A>G), rs1137100 (805A>G)
- **PPARA**: rs1800234 (Val227Ala),
- **Adiponutrin (PNPLA3)**: rs12483959(+1227G>A,), rs738409(444C>G)
- **Adiponectin (ADIPOQ)**: rs2241766 (+45T>G), rs1501299 (+276G>T), rs266729 (-11377C>G)

### Genotyping

Blood samples were collected in EDTA tubes and genomic DNA was prepared using the PUREGENE *DNA* isolation *kit* (*Genetra*, Minneapolis, MN, USA) according to user protocol. The SNPs were genotyped using a predesigned TaqMan SNP genotyping assay (Applied Biosystems, Foster City, CA, USA).

### Statistical analyses

All values are presented as mean±standard deviation. Normally distributed variables (age, gender, BMI, body fat, HbA1c, HOMA-IR, triglycerides, total cholesterol, HDL, LDL, AST, ALT, and HMW adiponectin) were compared between the patient and control groups using the t-test. Genotype and allelic frequencies were compared between the two groups by the Chi-Square test. Linear regression analysis was applied to estimate risk factors for NAFLD. One-way ANOVA was used to compare the relationship between serum adiponectin levels and different genotypes of rs266729. A p value less than 0.05 was considered statistically significant. Analyses were performed using the statistical package SPSS, version 13.0 (Chicago IL).

## Results

### Anthropometric and laboratory data

Table 1 shows the demographic and clinical data of patients with NAFLD (n = 350) and controls (n = 209). We found that patients with diabetes and fatty liver disease had significantly higher BMI values (27.1±5.2 vs. 23.1 ± 3.9 kg/m^2^), a higher percentage of body fat (28.2±5.6 vs. 23.2 ± 3.5%), higher triglyceride (155.4±92.8 vs. 120.5 ± 56.9 mg/dL) and ALT levels (35.4± 22.5vs. 24.2 ± 19.1 U/L), and lower HMW adiponectin levels (4.2±0.7 vs. 5.0±0.5 μg/ml) than DM patients without NAFLD (*p* ≦0.001 for all values). Total cholesterol and AST levels were moderatly higher in patients with NAFLD (183.6± 34.5 vs. 173.6± 31.2 mg/dL, *p* = 0.02; 29.1 ± 11.5 vs. 23.6 ± 12.6 U/L, *p* = 0.02, respectively). However, because of intensive therapy with different oral hypoglycemic agents and statins, there were no apparent differences in HbA1c, HDL or LDL levels between the two groups.

**Table 1:** Demographic and clinical data of the subjects.

Characteristics	Fatty liver (N = 350)	No fatty liver (N = 209)	*p-value*
Age (year)	52.0 ± 9.9	51.4 ± 10.6	*0*.*31*
Sex (female/male)	187/163	123/86	
BMI (kg/m2)	27.1 ± 5.2	23.1 ± 3.9	*<0*.*001*
Body fat (%)	28.2± 5.6	23.2 ± 3.5	*<0*.*001*
HbA1c (%)	7.88 ± 1.21	7.64± 1.13	*0*.*50*
HOMA-IR	7.01± 2.65	5.45 ± 2.23	*0*.*06*
Triglyceride (mg/dL)	155.4 ±92.8	120.5 ± 56.9	*<0*.*001*
TC (mg/dL)	183.6± 34.5	173.6± 31.4	*0*.*02*
HDL- (mg/dL)	55.5 ± 12.2	56.9 ± 14.8	*0*.*33*
LDL- (mg/dL)	96.4 ± 19.1	92.6 ± 30.1	*0*.*20*
AST (U/L)	29.1± 11.5	23.6 ± 12.6	*0*.*02*
ALT (U/L)	35.4± 22.5	24.2± 19.1	*<0*.*001*
HMW adiponectin (μg/ml)	4.2±0.7	5.0±0.5	*<0*.*001*

ALT, alanine aminotransferase; AST, aspartate aminotransferase; BMI, body mass index;, HbA1c, glycated hemoglobin; HDL-C, high-density lipoprotein cholesterol; HMW adiponectin, high moleculae weight adiponectin; HOMA-IR, homeostasis model assessment-insulin resistance; LDL-C, low-density lipoprotein cholesterol; TC, Total cholesteral; TG, triglyceride

### Genotypes and allele frequencies of SNPs

The distribution of genotypes and the frequency of alleles in each SNP between patients with NAFLD and control subjects are shown in Tables 2–4. There were no significant differences in genotypic distribution or allelic frequency between the NAFLD and control groups with the exception of adiponectin rs266729. Adiponectin rs266729 polymorphism was associated with susceptibility to NAFLD (p = 0.001). Subgroup analysis revealed that the proportion of subjects with homozygous genotype GG was higher in patients with NAFLD (31%) than in controls (11%) and that the proportions of heterozygous CG and homozygous CC were higher in controls (37% and 52%, respectively) than in patients with NAFLD (33% and 36%, respectively). Single locus analysis in pooled subjects also revealed that the G allele was associated with NAFLD (47% vs. 29% p = 0.008).

**Table 2:** The Genotypes and Allele Distribution of the PPARPGC1A (rs3736265, rs3755863, and rs2290602) and the LEPR (rs1805096, rs1137101, and rs1137100) Gene Polymorphisms in Case (Type 2 DM with NAFLD) and Control Groups (Type 2 DM without NAFLD).

	Genotypes (%)			p	Alleles (%)		p
**rs3736265**	A/A	A/G	G/G	**0.565**	A	G	**0.414**
NAFLD (n = 294)	106 (36)	132 (45)	56 (19)		344(59)	244(41)	
Control (n = 167)	67(40)	72(43)	28(17)		206 (62)	128(38)	
**rs3755863**	A/A	A/G	G/G	**0.666**	A	G	**0.543**
NAFLD (n = 311)	56(18)	103(33)	152(49)		199(39)	309(61)	
Control (n = 188)	23(12)	65(35)	100(53)		111(30)	265(70)	
**rs2290602**	G/G	G/T	T/T	**0.338**	G	T	**0.188**
NAFLD (n = 302)	54(18)	100(33)	148(49)		208(34)	396(66)	
Control (n = 200)	20(10)	78(39)	102(51)		118(30)	282(70)	
**rs1805096**	A/A	A/G	G/G	**0.221**	A	G	**0.176**
NAFLD (n = 145)	3(2)	6(4)	136(94)		12(4)	278(96)	
Control (n = 98)	0(0)	10(10)	88((90)		10(5)	186(95)	
**rs1137101**	A/A	A/G	G/G	**0.091**	A	G	**0.090**
NAFLD (n = 293)	214(73)	53(18)	26(9)		481(82)	105(18)	
Control (n = 187)	168(90)	15(8)	4(2)		351(94)	23(6)	
**rs1137100**	A/A	A/G	G/G	**0.347**	A	G	**0.100**
NAFLD (n = 301)	271(90)	24(8)	6(2)		566(94)	36(6)	
Control (n = 198)	172((87)	22(11)	4(2)		366(92)	30(8)	

PPARPGC1A, peroxisome proliferator-activated receptor γ co-activator 1α; LEPR, leptin receptor; NAFLD, non-alcoholic fatty liver disease, DM, diabetes mellitus.

**Table 3:** The Genotypes and Allele Distribution of the PPARA rs1800234 and adiponutrin (rs12483959 and rs 738409) Gene Polymorphisms in Case (Type 2 DM with NAFLD) and Control Groups (Type 2 DM without NAFLD).

	Genotypes (%)			p	Alleles (%)		p
**rs1800234**	C/C	C/T	T/T	**0.777**	C	T	**0.482**
NAFLD (n = 327)	288(88)	39(12)	2(0)		615(94)	43(6)	
Control (n = 195)	168(86)	27(14)	0(0)		363(93)	27(7)	
**rs12483959**	A/A	A/G	G/G	**0.111**	A	G	**0.404**
NAFLD (n = 327)	69(21)	150(46)	108(33)		288(44)	366(56)	
Control (n = 195)	51(26)	78(40)	66(34)		180(46)	210(54)	
**rs 738409**	C/C	CG	G/G	**0.344**	C	G	**0.500**
NAFLD (n = 327)	114(35)	131(40)	82(25)		359(55)	295(45)	
Control (n = 195)	58(30)	82(42)	55(28)		198(51)	192(49)	

PPAR, peroxisome proliferator-activated receptor alpha; NAFLD, non-alcoholic fatty liver disease, DM, diabetes mellitus.

**Table 4:** The Genotypes and Allele Distribution of the adiponectin Gene Polymorphisms in Case (Type 2 DM with NAFLD) and Control Groups (Type 2 DM without NAFLD).

	Genotypes (%)			p	Alleles (%)		p
**rs2241766**	T/T	T/G	G/G	**0.098**	T	G	**0.218**
NAFLD (n = 350)	199(57)	130(37)	21(6)		528(75)	172(25)	
Control (n = 209)	129(62)	63(30)	17(8)		321(77)	97(23)	
**rs1501299**	G/G	G/T	T/T	**0.663**	G	T	**0.165**
NAFLD (n = 350)	175(50)	126(36)	49(14)		476(68)	224(32)	
Control (n = 209)	113(54)	79(38)	17(8)		305(73)	113(27)	
**rs266729**	C/C	C/G	G/G	**0.001**	C	G	**0.008**
NAFLD (n = 350)	126(36)	116(33)	108(31)		368(53)	332(47)	
Control (n = 209)	109(52)	77(37)	23(11)		295(71)	123(29)	

NAFLD, non-alcoholic fatty liver disease, DM, diabetes mellitus.

### Predictors of NAFLD

Multiple linear regression analyses were performed to assess the factors predictive of NAFLD in patients with type 2 DM. The markers included BMI, body fat, HOMA-IR, TG, TC, HDL, AST, ALT, and HMW adiponectin (Table 5). The results showed that ALT, body fat percentage, and serum HMW adiponectin levels are strong predictors of NAFLD in patients with type 2 diabetes (p = 0.007,< 0.001 and 0.004, respectively). TC levels was moderate predictor of the disease in patients with type 2 diabetes (p = 0.017).

**Table 5:** Independent effects of markers of metabolic control on non-alcoholic fatty liver disease.

variable	Standardized Coefficients	T	Sig.
**BMI**	.005	0.52	.910
**Body fat**	.312	3.548	.000
**HbA1c**	-.006	-.128	.784
**TG**	.004	.050	.930
**TC**	.355	2.389	.017
**LDL**	-.271	-.2.023	.038
**HDL**	-.142	-1.719	.099
**ALT**	.159	2.659	.007
**AST**	-.060	-.492	.412
**HMW Adiponectin**	-.138	-2.871	.004

BMI, Body mass index, HbA1c, glycated hemoglobin; HDL-C, high-density lipoprotein cholesterol; LDL-C, low-density lipoprotein cholesterol; TC, total cholesteral; TG, triglyceride; AST, aspartate aminotransferase, ALT, alanine aminotransferase; HMW adiponectin, high moleculae weight adiponectin. Dependent variable: fatty liver or fatty liver. Predictors: BMI, %body fat, HbA1c, TG, TC, LDL, HDL, ALT, AST and HMW adiponectin

### Relationship between serum adiponectin levels and different genotypes of SNP rs266729 (Fig 1)

**Fig 1:**
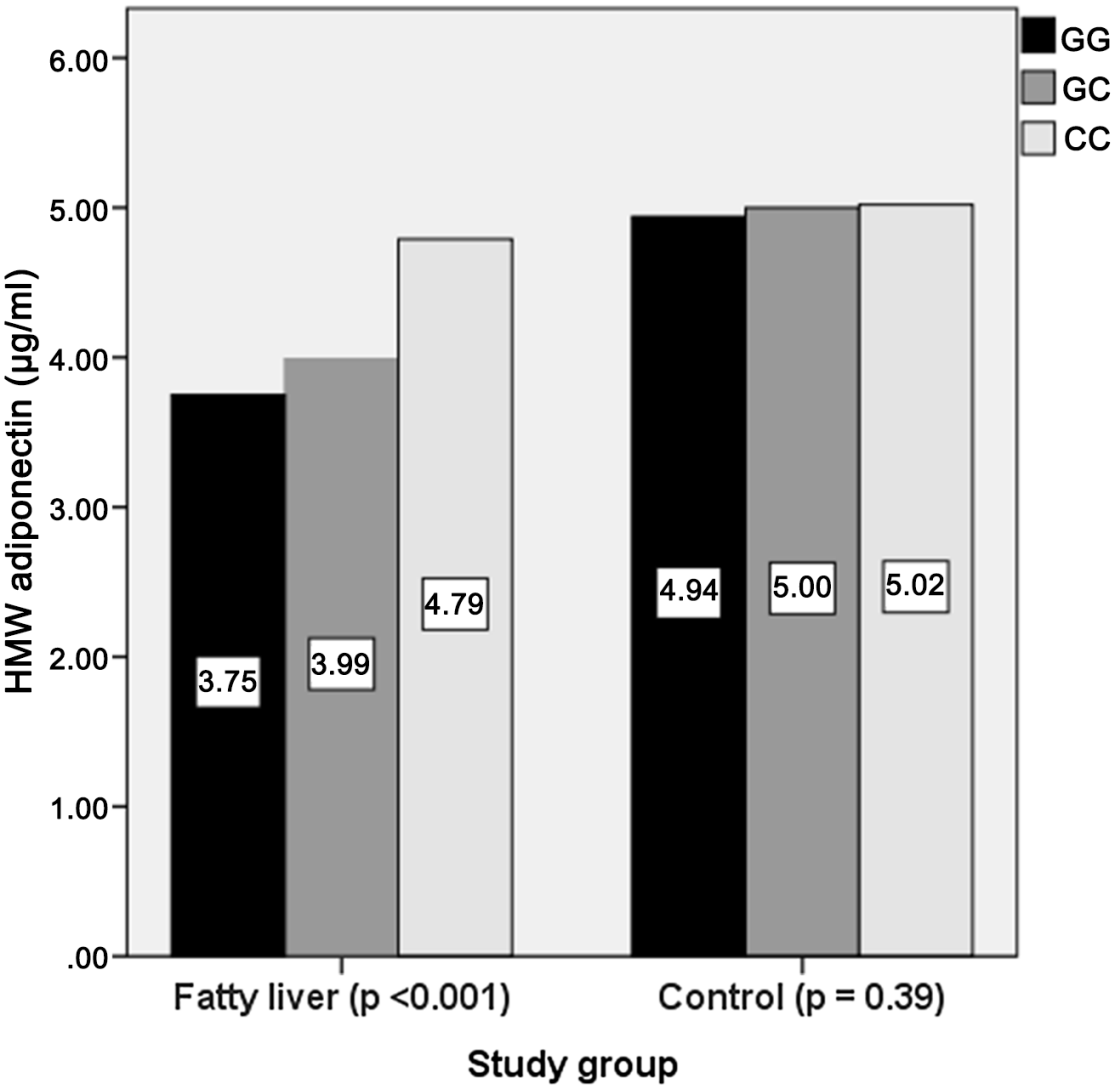
Plasma HMW adiponectin levels in the study groups. Median plasma HMW adiponectin levels were compared between GG, GC and CC genotypes of SNP rs266729 in patients with non-alcoholic fatty liver disease and control.

Patients with NAFLD had significantly lower HMW adiponectin levels than controls (4.2±0.7 vs. 5.0±0.5 μg/ml, p< 0.001). In addition, patients with type 2 diabetes and NAFLD who carried the GG genotype of rs266729 had significantly lower serum HMW adiponectin levels than patients with NAFLD who carried the GC or CC genotype (3.75±0.37 vs.3.99±0.66 vs. 4.79±0.58 μg/ml, p< 0.001). In patients with type 2 diabetes but without NAFLD, there was no significant difference in plasma HMW adiponectin levels between patients with different genotypes (GG: 4.94±0.44 vs. GC: 5.00±0.54 vs. CC:5.02±0.30μg/ml, p = 0.39).

## Discussion

In this study, we investigated the possible association between multiple published candidate gene polymorphisms and NAFLD in a group of patients with type 2 diabetes in Taiwan. We found that adiponectin gene rs266729 polymorphism was associated with an increased risk of NAFLD in patients carrying the GG genotype. A number of studies in different populations have demonstrated that adiponectin gene polymorphisms influence the development of NAFLD [23–29]. To the best of our knowledge, few studies have investigated the role of gene polymorphisms on the development of NAFLD in patients with type 2 diabetes. Tokushige et al. found that the frequency of G/G in patients with the rs1501299 (+276 G>T) of adiponectin trended to be higher in NAFLD patients than in controls, but not significant. In this study, they also found that adiponectin rs2241766 (+45 T>G) genotype G/G was associated with the progression of liver fibrosis in Japanese with NAFLD [24]. However, in their study less than half of the patients (44.5%) had diabetes mellitus and the association between rs1501299 and NAFLD was only significant in female patients. Zhou et al. reported that the adiponectin SNP rs1501299 (+276 G>T) was associated with the development of NAFLD in Chinese. However, their study did not include patients with diabetes and their patients had lower HOMA-IR threshold values than patients in our study [25]. Another study revealed that adiponectin rs2241766 (+45 T>G) and rs1501299 (+276 G>T) were not important determinants of NAFLD but still influenced other components of metabolic syndrome in Chinese [23]. Furthermore, the T/T genotype of adiponectin gene rs1501299 has been shown to be associated with a high risk of developing cardiovascular disease [30].

The relationship between obesity and some types of cancer has shown to be mediated by the SNP rs266729 (-11377 C>G) [31]. Although Hashemi et al. found a significant difference in the frequency of the rs266729 polymorphism between patients with and those without NAFLD, they did not specifically recruit patients with type 2 diabetes [29]. In Thai study, decreased concentration of adiponectin is associated with rs266729 (-11377C>G) polymorphism and this polymorphism of the adiponectin gene is significantly more frequent in the patients with metabolic syndrome [32]. In our current study, we genotyped 3 SNPs of adiponectin (rs266729, rs1501299 and 2241766). We also found that there was a significant difference in frequency of adiponectin rs266729 but not rs1501299 or 2241766 gene polymorphisms between patients with and those without NAFLD. This was also confirmed by a recent meta-analysis revealed that adiponectin gene rs17300539 and rs1501299 polymorphisms are associated with an increased risk of obesity in Caucasians and that the rs266729 polymorphism is associated with risk of obesity in Asians and there is no associations between rs2241766 and the obesity risk [31].

Adiponectin is an adipocytokine and a hepatic insulin sensitizer that plays an important role in the pathogenesis of DM. Adiponectin stimulates fatty acid oxidation and decreases insulin resistance by attenuating the accumulation of triglycerides in serum. In a previous study, we found that hypoadiponectinemia was associated with NAFLD, even in patients without DM and obesity [33]. Hui et al. reported similar findings about adipokines (adiponectin, adipocyte fatty acid binding protein and fibroblast growth factor-21) in the pathophysiology of NAFLD and diabetes [34]. In the present study we found that serum levels of adiponectin were negatively associated with NAFLD and that percentage of body fat, BMI, and serum levels of TG and ALT were positively associated with NAFLD. In addition to percentage of body fat, plasma levels of adiponectin seem to be better predictors of NAFLD than other biomarkers of metabolic syndrome. Similar results have also been reported [35,36]. In patients with diabetes but without NAFLD, we did not observe any significant difference on serum levels of adiponectin in patients with different genotypes. Among patients with diabetes and NAFLD, we found that serum adiponectin levels were lower in patients with the GG genotype than in patients with the CG or CC genotype. Our results suggest that the G/G variant of adiponectin gene rs266729 is associated with hypoadiponectinemia and susceptibility to NAFLD. Serum adiponectin may be a protective factor to prevent patients with diabetes to be complicated with NAFLD. That’s further proving that adiponectin is a protective adipocytokines. Increasing serum adiponectin levels may prohibit liver steatosis. The potential use of adiponectin as a therapeutic target in patients with type 2 diabetes complicated with NAFLD may be feasible.

We also genotyped 3 SNPs of the PPARGC1A gene, 3 SNPs of the LEPR gene, 1 SNP of PPARA and 2 SNPs of PNPLA3 to measure the association of those SNPs with NAFLD. We also compared the frequency of the genotypes and alleles of those SNPs, between patients with and those without NAFLD. Association between PPARGC1A polymorphisms and the occurrence of nonalcoholic fatty liver disease was first demonstrated in Yoneda et al’s study [15]. They found that rs2290602 was significantly associated with NAFLD and T allele of rs2290602 was significantly higher in the nonalcoholic steatohepatitis patients. The rs3736265 and rs3755863 of PPARGC1A gene also had significant allele frequency in Yoneda’s study but not significant after conservative Bonferroni's correction was applied. The variant frequency at nucleotide 3057 G>A (rs1805096) of LEPR was 76.0% in type 2 diabetic patients complicated with NAFLD but only 104 patients enrolled [13]. Association between variants of LEPR rs1137100 and rs1137101 with risk of NAFLD was also revealed in Asian group but only G allele of t rs1137100 was associated with a less severe form of liver disease in patients with NAFLD [14]. In Chen et al’s report, there were 93.67% subjects with TT genotype of rs1800234 (PPARA val227val) in patients with NAFLD and to be a protective role in obesity patients. The parameters of metabolic syndrome in subjects with Val227Ala variant were significantly lower than that in Val227wide type [16]. In obese Taiwanese children, PNPLA3 rs738409 was revealed to have association with an increased risk of NAFLD [18]. The G allele in PNPLA3 rs738409 increases the risk of NAFLD in the general population, especially in subjects without metabolic syndrome [19]. Our results revealed that in patients with type 2 diabetes, there was no significant differencesin these gene polymorphisms between patients with and without NAFLD. The difference between our results and results reported in previous studies in Asian populations may be because all of our enrolled patients all had type 2 diabetes. NAFLD having higher prevalence in patients with type 2 diabetes may have different gene polymorphyism from patient without type 2 diabetes. Although Lu et al. showed that polymorphisms of the LEPR gene G3057A (rs1805096) probably contribute to the onset of NAFLD by regulating lipid metabolism and affecting insulin sensitivity in patients with type 2 diabetes, the researchers only used ultrasound to determinate the presence of fatty liver [13]. In our study, we used elastography to accurately confirm the presence of steatosis. Although liver biopsy is the gold standard to evaluate fatty liver, biopsy is not appropriate for large-scale clinical studies. Elastography, on the other hand, is a non-invasive method that can accurately and semiquantitatively measures the fat content in liver [37].

In Taiwanese patients with type 2 diabetes, gene polymorphism of adiponectin rs266729 is associated with risk of NAFLD. G allele of rs266729 is associated with hypoadiponectinemia. Low serum adiponectin level may precipitate liver steatosis in patients with type 2 diabetes. Serum adiponectin levels may be a target therapeutic marker of fatty liver in patients with type 2 diabetes.
